# 15-Lipoxygenase Metabolites of Docosahexaenoic Acid Inhibit Prostate Cancer Cell Proliferation and Survival

**DOI:** 10.1371/journal.pone.0045480

**Published:** 2012-09-20

**Authors:** Joseph T. O’Flaherty, Yungping Hu, Rhonda E. Wooten, David A. Horita, Michael P. Samuel, Michael J. Thomas, Haiguo Sun, Iris J. Edwards

**Affiliations:** 1 Department of Internal Medicine_,_ Wake Forest School of Medicine, Winston-Salem, North Carolina, United States of America; 2 Department of Pathology, Wake Forest School of Medicine, Winston-Salem, North Carolina, United States of America; 3 Department of Biochemistry_,_ Wake Forest School of Medicine, Winston-Salem, North Carolina, United States of America; Fundação Oswaldo Cruz, Brazil

## Abstract

A 15-LOX, it is proposed, suppresses the growth of prostate cancer in part by converting arachidonic, eicosatrienoic, and/or eicosapentaenoic acids to n-6 hydroxy metabolites. These metabolites inhibit the proliferation of PC3, LNCaP, and DU145 prostate cancer cells but only at ≥1–10 µM. We show here that the 15-LOX metabolites of docosahexaenoic acid (DHA), 17-hydroperoxy-, 17-hydroxy-, 10,17-dihydroxy-, and 7,17-dihydroxy-DHA inhibit the proliferation of these cells at ≥0.001, 0.01, 1, and 1 µM, respectively. By comparison, the corresponding 15-hydroperoxy, 15-hydroxy, 8,15-dihydroxy, and 5,15-dihydroxy metabolites of arachidonic acid as well as DHA itself require ≥10–100 µM to do this. Like DHA, the DHA metabolites a) induce PC3 cells to activate a peroxisome proliferator-activated receptor-γ (PPARγ) reporter, express syndecan-1, and become apoptotic and b) are blocked from slowing cell proliferation by pharmacological inhibition or knockdown of PPARγ or syndecan-1. The DHA metabolites thus slow prostate cancer cell proliferation by engaging the PPARγ/syndecan-1 pathway of apoptosis and thereby may contribute to the prostate cancer-suppressing effects of not only 15-LOX but also dietary DHA.

## Introduction

The metabolism of dietary fatty acids is of particular interest in prostate cancer, the most frequently diagnosed cancer and a leading cause of death in American males. Epidemiological studies suggest that intake of the n-3 marine polyunsaturated fatty acids (PUFA), eicosapentaenoic acid (EPA, 20∶5, n-3) and docosahexaenoic acid (DHA, 22∶6, n-3) reduces prostate cancer risk [1], [2]. Moreover, tissue levels of n-3 PUFA were inversely associated with prostate cancer progression [3], [4], [5]. In addition, cell culture and animal models have shown that n-3 PUFA are protective whereas n-6 PUFA promote this cancer [6], [7], [8]. PUFA are incorporated into cell membrane phospholipids and are substrates for oxygenase (cyclooxygenase and lipoxygenase) enzymes to be metabolized to bioactive lipids. One proposed mechanism for the tumor-inhibitory activity of n-3 PUFA is competitive inhibition of the oxygenases used by n-6 PUFA to form tumor-promoting metabolites (reviewed in [9], [10]). Our studies [11], [12] and those of others [6], [13] have shown that DHA is a strong inhibitor of prostate cancer cell growth, a property that is regulated by a 15-lipoxygenase (15-LOX) (unpublished studies).

Previous studies have indicated that two isoforms of 15-LOX identified in humans may play opposing roles in the development and progression of prostate cancer through metabolism of n-6 PUFA. 15-LOX-1 is more highly expressed in malignant than normal human prostate tissue and its levels correlate positively with the disease’s severity [14], [15], [16]. It prefers linoleic acid (LA) to arachidonic acid (AA) and in consequence makes mainly the LA metabolite, 13-hydroxy-octadecaenoic acid (HODE) [14], [17], [18]. Prostate cancer has higher levels of 13-HODE and converts LA to 13-HODE to a greater extent than normal prostate tissue [14], [15], [16]. 15-LOX-2, in contrast, prefers AA over LA, makes mainly the AA metabolite, 15-hydroxy-eicosatetraenoic acid (15-HETE), is under-expressed or absent in prostate cancer, and its levels correlate negatively with disease severity [14], [17], [18], [19], [20]. Human prostate cancer has relatively little ability to convert AA to 15-HETE [15]. Experimental studies have supported and expanded these clinical observations.

Mice made to express in their prostate glands human 15-LOX-1 develop prostate intraepithelial neoplasia (PIN) [21]; when similarly engineered to express human 15-LOX-2, they develop prostates enlarged with senescent cells [22]. Correlating with these results, the forced expression of human 15-LOX-1 speeds and 15-LOX-1 knockdown slows the proliferation of cultured and explanted human prostate cancer cells [23]. Forced expression of human 15-LOX-2 causes these cells to stop proliferating and become senescent [24], [25]. The effect of 15-LOX-1 appears due to its production of 13-HODE, which enhances the ability of growth factors to stimulate prostate cancer cell proliferation [23], [26], [27]. The effect of 15-LOX-2 is attributed in part to its production of 15-HETE, which inhibits prostate cancer cell proliferation [24], [25], [26] through activation of peroxisome proliferator-activating receptor (PPAR)-γ [26], [28], [29]. These results suggest that the progression of prostate epithelial cells into malignancy involves up-regulating 15-LOX-1 and down-regulating 15-LOX-2 to create an environment favoring growth, i.e. one richer in pro-proliferative and poorer in anti-proliferative PUFA metabolites. There are issues with this 15-LOX/n-6 PUFA model. AA itself causes prostate cancer cells to proliferate [30] and like other n-6 PUFA is suggested to promote rather than suppress prostate cancer in some epidemiology studies [31], [32], [33]. Moreover, the anti-proliferative action of 15-HETE on cultured prostate cancer cells requires ≥10–100 µM [25], [26], [34]. The corresponding major metabolites of the n-6 PUFA, γ-linolenic acid (GLA), and the n-3 PUFA, eicosapentaenoic acid (EPA), are also less likely mediators of 15-LOX-2′s anti-cancer effect since both 15-hydroxy-eicosatrienoic and 15-hydroxy-eicosapentaenoic acid require ≥1–5 µM to slow the proliferation of prostate cancer cells [35], [36]. The existing data thus warrant searches for other 15-LOX/PUFA metabolite models.

We here examine the activity, potency, and mechanism of action of 15-LOX metabolites of DHA. These metabolites are of particular interest because 1) DHA is a member of the n-3 PUFA family suggested to suppress prostate cancer in epidemiological studies [31], [32], [33]; 2) DHA is a key contributor to the anti-proliferative effect of n-3 PUFA in prostate cancer cells [11], [12] and 3) the activity of shorter chain n-3 PUFA, including EPA, may be irrelevant to DHA’s activity since men [37] as well as cultured prostate cancer cells [11] can readily convert shorter chain n-3 PUFA to EPA but are virtually incapable of converting EPA to DHA.

## Materials and Methods

DHA and AA (NuChek Prep); soybean 15-LOX type 1a (sLOX), sodium borate, and sodium borohydride (Sigma); HPLC columns (Waters); HPLC or optima grade organic solvents and diethyl ether (Fisher); anti-SDC-1 (H-174) antibody (Santa Cruz Biotechnology, Inc); anti-HRP-conjugated secondary antibody against rabbit antibody (Cell Signaling Technology); and CellTiter 96® Aqueous One Solution Cell Proliferation Assay and Caspase-Glo® 3/7 Assay (Promega) were purchased. PC3, DU145, and LNCaP human prostate cancer cell lines (American Type Culture Collection (Manassas, VA) were grown in advanced Dulbecco’s modified Eagle medium (Invitrogen) containing 1% fetal bovine serum (PC3 cells), Eagle’s minimum essential medium with Earle’s salts medium (Invitrogen) containing 10% fetal bovine serum (DU145 cells), or RPMI 1640 medium (Invitrogen) with 10% fetal bovine serum (LNCaP cells) as described [34], [38].

### Metabolite Preparations

We prepared 15S-hydroperoxy-eicosatetra-5Z,8Z,11Z,13E-enoic (15-HpETE), 15S-hydroxy-eicosatetra-5Z,8Z,11Z,13E-enoic (15-HETE), 5S,15S-dihydroxy-eicosa-tetra-6E,8Z,11Z,13E-enoic (5,15-diHETE), and 8S,15S-dihydroxy-eicosatetra-5Z,9E,11Z, 13E-enoic (5,15-diHETE) acids by reacting arachidonic acid (AA) with sLOX [39] and used this same method to prepare DHA metabolites. Briefly, DHA (10^−4^ M) was reacted with 0.8 mg of sLOX in 50 ml of aerated sodium borate buffer (50 mM; pH 9; 4°C, 30 min). Reactions were extracted with diethyl ether; the 17S-hydroperoxy-docosa-hexa-4Z,7Z,10Z,13Z,15E,19Z-enoate (17-HpDHA) product was purified by gravimetric silicic acid chromatography, isocratic C18 µ-Bondapak HPLC (1.5×300 mm; methanol:H_2_O:glacial acetic acid, 750/250/0.1, v/v; 3 ml/min; eluting at ∼34 min), and isocratic μ-Porasil HPLC (1.5×300 mm; hexane:isopropanol:glacial acetic acid, 950∶50:1, v/v; 5 ml/min; eluting at ∼6 min). Elution UV spectra were monitored with a G1315A diode array spectrometer ran with ChemStation 51 software (Agilent Technologies). 17-HpDHA was reacted with sodium borohydride in methanol and re-purified by μ-Bondapak HPLC to obtain 17S-hydroxy-docosahexa-4Z,7Z,10Z,13Z,15E,19Z-enoate (17-HDHA). For dihydroxy products, DHA (10^−4^ M) in 500 ml reactions was reacted with 10 mg of sLOX added at 0, 45, 90, 150, and 240 min. After 300 min, the reaction was processed like 17-HpHDA though the μ-Bondapak HPLC step; the peak eluting in this system at ∼10 min with a triene absorbance spectra (maxima: 280, 270, and 261 nm) dominating its left side and 5,15-diHETE-like absorbance spectra (maximum: 243; adiabatic hump: ∼223 nm) dominating its right side was collected; reduced with sodium borohydride; and, following Butovich et al. [40], [41], resolved by isocratic 5SW HPLC (3×250 mm; hexane: isopropanol:glacial acetic acid (974∶26:1, v/v; 1 ml/min) into peaks at ∼17 and 22 min with respective UV spectra for 10S,17S-dihydroxy-docosahexa-4Z,7Z,11E,13Z,15E,19Z-enoate (10,17-diHDHA also termed protectin DX [42]; maxima: 280, 270, and 260 nm) and 7S,17S-dihydroxy-docsahexa-4Z,8E,10Z,13Z,15E,19Z-enoate (7,17-diHDHA [or protectin D5]; maximum: 222 nm; adiabatic hump: 242 nm) [40], [41], [43]. In addition to their HPLC elution times and UV spectra, the structures of the AA metabolites were confirmed by MS [39] and of the DHA metabolites by MS and nuclear magnetic resonance (NMR). 17-HDHA and 10,17-diHDHA gave electrospray spectra (Quattro II MS, MassLynx 3.5 software, negative ion mode) similar to those published [40], [41], [43], [44], [45], [46]. The molecular ion for 17-HpDHA was 16 AMU greater than that for 17-HDHA. NMR spectra (1D and 2D double-quantum-filtered COSY in d_4_-methanol; 25°C; Bruker 699 MHz Avance NMR spectrometer) for 17-HDHA and 10,17-diHDHA had chemical shifts and coupling patterns matching published reports [40], [41], [44]; the deduced conjugated double bond geometries were for 17-HDHA, 13Z,15E; for 10,17-diHDHA, 11E,13Z,15E; and for 7,17-diHDHA, 8E,10Z and 13Z,15E. The four DHA metabolites lacked resonances at 6.1–6.2 ppm indicating the absence of a trans-trans conjugated double bond. The PUFA and metabolites were stored in methanol under an argon atmosphere at −80°; freed of methanol by a stream of nitrogen; taken up in culture media; and added to cell cultures. Due to their instability [40], [41], 15-HpETE and 17-HpDHA were used within 3 weeks of preparation.

### Proliferation and Caspase Assays

Proliferation was assayed with Cell Titer96 Aqueous One Solution Cell Proliferation Assays (Promega) as described [47]. To measure apoptotic activity, cells were seeded in 96-well plates at a density of 1000 cells/well for 24 h, then treated with the compounds for 48 h prior to measurement of caspase activity using the Caspase-Glo® 3/7 assay (Promega) according to the manufacturers directions.

### PPARγ Activation Assay

2×10^5^ PC3 cells were seeded on 35 mm dishes in 1 ml of advanced DMEM with 1% FBS, for 24 h and transfected with 1 µg of lacZ and 1 µg of PPRE DNA (PPAR response element-luciferase reporter) [34], [38] using FuGENE 6 Transfection Reagent (Roche). In some studies, cells were co-transfected with a vector (1 µg) encoding dominant negative (d/n)-PPARγ (L468/E471) [34] [38] or empty vector pcDNA3 (Invitrogen, Carlsbad, CA) for 24 h or were treated for 30 min with the PPARγ antagonist, GW9662. The cells were then challenged with a DHA metabolite or a PPARγ agonist, troglitazone, for 24 h. Cells were scraped into Reporter Lysis Buffer (Promega). Samples were frozen for 18 h and centrifuged (200 g, 4 min, 20−C). Supernatant fluids were assayed for luciferase and β-galactosidase (Promega Luciferase and β-Galactosidase Enzyme Assay Systems). Luciferase was corrected for transfection efficiency based on β-galactosidase as in [34], [38].

### SDC-1 Assay

To detect SDC-1 message, total PC3 cell RNA was prepared and amplified in triplicate using the Applied Biosystems 7500 Real-Time PCR System. Primers for human SDC-1 were 5′-ggagcaggacttcacctttg (forward) and 5′-ctcccagcacctctttcct (reverse). Data were normalized to the housekeeping control peptidyl-prolylisomerase B and are presented as relative to control. To detect SDC-1 protein, PC3 cells were homogenized and lysed in ice-cold buffer (25 mM Tris-HCl, 150 mM NaCl, 1% Triton X-100, 0.1 mg/ml phenyl-methanesulfonyl fluoride, 1× proteinase, and 1× phosphatase inhibitors [Roche Applied Science]), dialyzed against 100 mM Tris and 30 mM sodium acetate, pH 8.0, for 24 h at 4°C, and digested by chondroitinase ABC (Seikagaku, Ijamsville, MD) and heparinase III (Sigma-Aldrich) at 37°C overnight. Protein extracts were prepared for Western blot analysis as described using the indicated antibody [12]. Band densities on photographic films were analyzed using Image J 1.37v (National Institutes of Health, Bethesda, MD). To silence SDC-1, 1×10^5^ PC3 cells per well were plated in 96-well plates, transfected with a small interfering RNA (siRNA) for the human SDC-1 gene (Ambion, catalog no. AM16708) or a negative control siRNA with no known target using Lipofectamine 2000 (Invitrogen) to achieve a knockdown efficiency of >75% as described [12]. Cultures were incubated for 18 h and then challenged with a DHA metabolite for 3 days.

### Statistical Analyses

Data are expressed as mean ± SD when data shown are from one experiment that was repeated with similar results or SEM where results are shown as the mean of independent experiments. Results were analyzed by ANOVA (one way or two way as indicted) and Bonferroni’s Multiple Comparison post test using GraphPad Prism version 4.00 for Windows, GraphPad Software, San Diego CA. Differences were considered significant at *P*<0.05.

## Results

**Figure 1:**
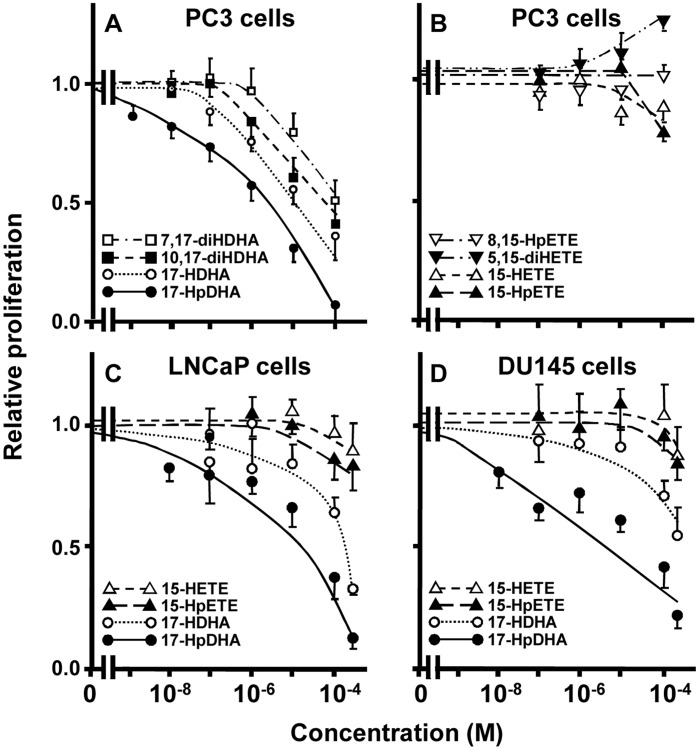
The proliferation responses of PC3, LNCaP, and DU145 prostate cancer cells to selected DHA and AA metabolites. The indicated cell types were incubated for 3 days with the indicated metabolite and their proliferation presented as the mean ± SEM (≥3 independent experiments) fractions of that found in cells treated with the vehicle (culture media) for the metabolites.

17-HpDHA, 17-HDHA, 10,17-diHDHA, and 7,17-HDHA slowed the proliferation of PC3 cells by 25% at about 0.1, 1, 8, and 10 µM, respectively (Fig. 1A). Under the same conditions, DHA required >60 µM to achieve this effect [12] and the analogous AA-derived 15-LOX metabolites, 15-HpETE, 15-HETE, and 8–15-diHETE, had far less or no activity while 5,15-diHETE slightly stimulated proliferation (Fig. 1B). The comparable 15-hydroxy metabolites of EPA and GLA reportedly require ≥5 µM to slow proliferation by 25% [35], [36] while the 15-LOX-dependent metabolites of LA, 13-HODE and 9-HODE, lacked anti-proliferative activity at <100 µM and actually stimulated proliferation at ≥0.1 and 1 nM, respectively (unpublished observations). 17-HpDHA and 17-HDHA also proved more potent than 15-HpETE or 15-HETE in slowing the proliferation of LNCaP and DU145 prostate cancer cells (Fig. 1C and 1D). Similar results occurred in the three cell lines when incubated with the metabolites for 48 or 96 h (results not shown). Under identical conditions, DHA required ≥30 µM to inhibit the proliferation of these cells [12].

**Figure 2:**
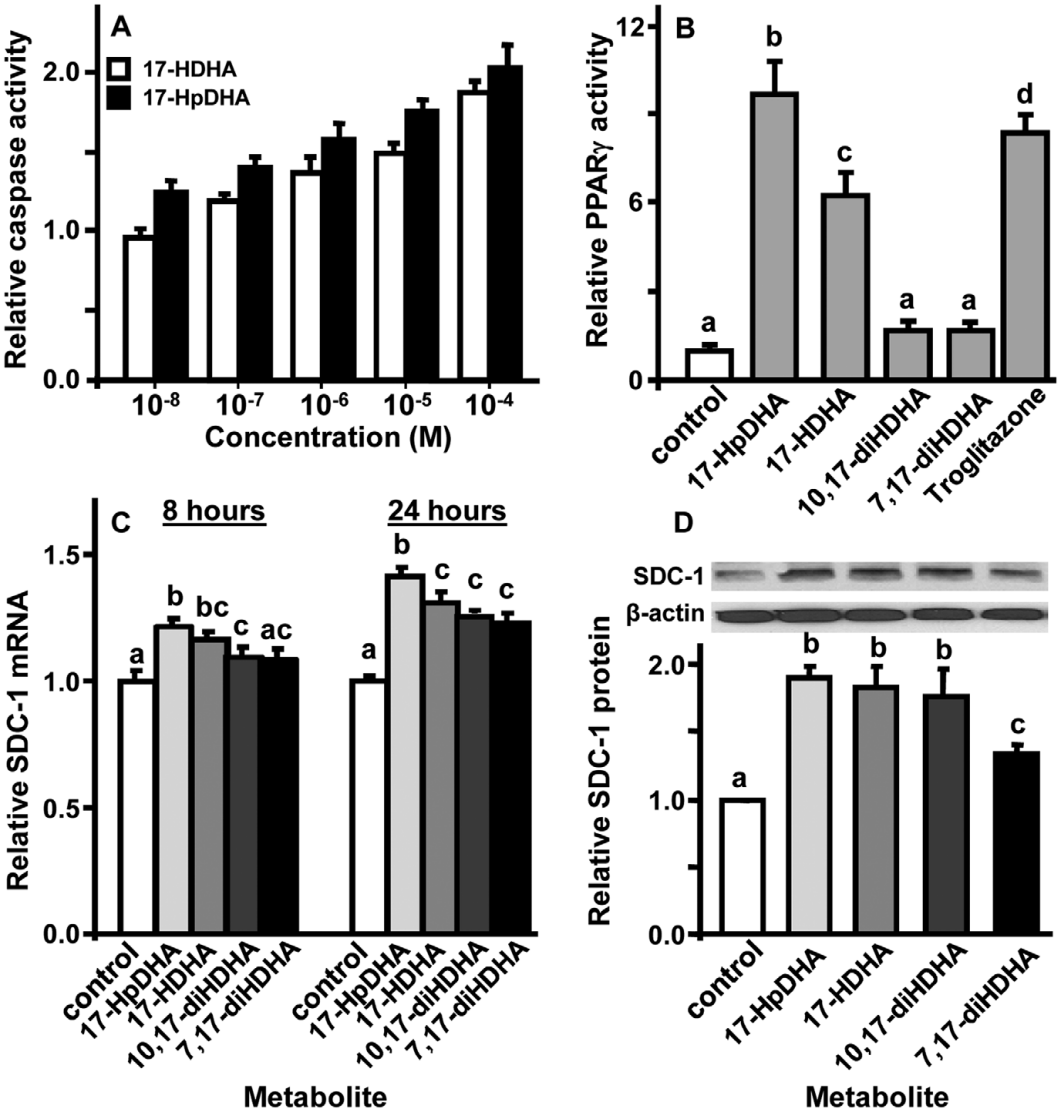
Stimulatory effects of DHA metabolites on caspase, PPARγ activity, and SDC-1 expression in PC3 cells. **A.** Cells were incubated with the indicated concentration of 17-HDHA or 17-HpDHA for 24 h and caspase-3 activity was measured by Caspase-Glo® 3/7 assay. Results are presented as mean ± SD (N = 3) relative to control cells treated with the medium for the metabolites. Responses to all doses at and above 10^−8^ M for 17-HpDHA and at or above 10^−7^ M for 17-HDHA were significantly greater than that of control cells (two way ANOVA, P<0.05). **B.** Cells transfected with luciferase PPARγ reporter gene were stimulated for 24 h with 10 µM of the indicated metabolite (the lowest dose where all had a clear effect on cell growth) or 5 µM of troglitazone and assayed for luciferase. Values represent the mean ± SD (N = 3). Bars labeled with the same letters are not significantly different from each other; bars labeled with different letters are significantly different from each other (one-way ANOVA, P<0.05) **C.** Cells were treated with medium (control) or 10 µM of the indicated metabolite for 8 or 24 h and their SDC-1 mRNA was measured. Values represent the mean, ± SD (N = 3). Within each time group, bars labeled with the same letters are not significantly different from each other; bars labeled with different letters are significantly different from each other (one-way ANOVA, P<0.05). **D.** Cells were treated with medium (control) or 10 µM of the indicated metabolite for 72 h and their lysates were analyzed for SDC-1. The Western blot is representative of 3 independent experiments. Values in graphs represent the mean ± SEM (N = 3 independent experiments). Bars labeled with different letters are significantly different from each other (one-way ANOVA, P<0.05).

To examine the mechanism(s) underlying the metabolites’ anti-proliferative activity, we focused on PC3 cells and followed earlier studies which found that DHA inhibits prostate cancer cell proliferation by activating PPARγ to induce the expression of syndecan (SDC)-1. This transmembrane proteoglycan has apoptosis-inducing activity for prostate and other cancer cells [12], [38], [48], [49]. 17-HpDHA and 17-HDHA, at ≥0.01 and 1 µM, respectively, caused PC3 cells to activate caspase-3, a marker for apoptosis (Fig. 2A). Statistical analysis (two way ANOVA) indicated that the dose-response variable significantly (p<0.0001) and the two metabolite variable significantly (p<0.001) impacted the results with 17-HpDHA being more potent that 17-HDHA. These same two DHA metabolites also caused the cells to activate a PPARγ reporter gene; this effect was similar to that of the pharmacological PPARγ activator, troglitazone (Fig. 2B), as well as DHA [38], [48]. Both 10,17-diHDHA and 7–17-HDHA were weak activators of PPARγ in this study inducing respectively a 69% and 68% increase in activity over control that trended toward but did not achieve statistical significance (P<0.1) (Fig. 2B). We have previously shown that both 15-HETE and 5,15-diHETE (1–200 µM) failed to activate this reporter [34]. Finally, the DHA metabolites stimulated PC3 cells to express SDC-1 mRNA (Fig. 2C) that resulted in increased SDC-1protein (Fig. 2D); these effects also matched those of troglitazone and DHA [12], [38], [48], [49]. The relative potencies of the metabolites in producing these responses approximated their relative potencies in slowing PC3 cell proliferation. The discrepancy between weak activation of PPARγ by 10,17-diHDHA and 7–17-HDHA (Fig. 2B) and their more robust effect on the accumulation of SDC-1 protein over 72 h (Fig. 2D) may suggest a slower uptake and/or metabolism of these two more polar products by the cells.

**Figure 3:**
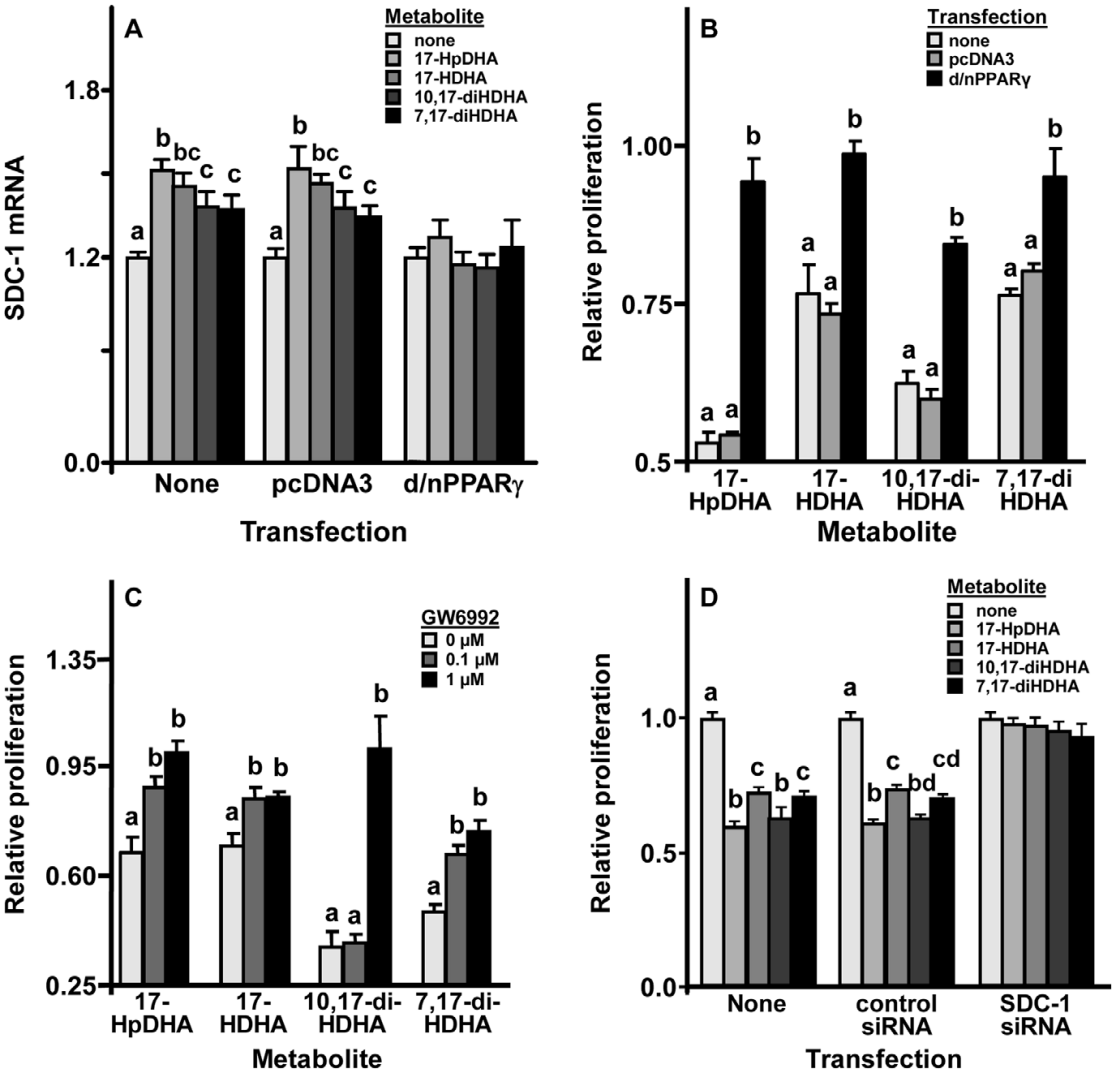
Inhibition of the effects of DHA metabolites in PC3 cells. **A.** Cells not transfected or transfected with pcDNA3 or d/nPPARγ were challenged with 10 µM of the indicated metabolite for 24 h before assaying syndecan-1 mRNA. Values represent the mean ± SD (N = 3). Within a transfection group, bars labeled with the same letters are not significantly different from each other; bars labeled with different letters are significantly different from each other (one-way ANOVA, P<0.05). **B.** Cells untransfected or transfected with pcDNA3 or d/nPPARγ were challenged with 10 µM of the indicated metabolite for 3 days before assaying proliferation. Values represent the mean ± SD (N = 3). Within a metabolite group, bars labeled with the same letters are not significantly different from each other; bars labeled with different letters are significantly different from each other (one-way ANOVA, P<0.05). **C.** Cells were incubated with 0–1 µM of PPARγ antagonist, GW6692, for 30 min and with 10 µM of the indicated metabolite for 3 days before assaying proliferation. Results are presented as mean ± SEM (N = 3 independent experiments). Within a metabolite group, bars labeled with the same letters are not significantly different from each other; bars labeled with different letters are significantly different from each other (one-way ANOVA, P<0.05). **D.** Cells untransfected, transfected with control siRNA, or transfected with SDC-1 siRNA were challenged with 10 µM of the indicated metabolite for 3 days before assaying proliferation. Results are presented as mean ± SD (N = 4). Within a transfection group, bars labeled with the same letters are not significantly different from each other; bars labeled with different letters are significantly different from each other (one-way ANOVA, P<0.05).

PPARγ activation appeared critical for the activity of the DHA metabolites: the metabolites were markedly inhibited from inducing SDC-1 in PC3 cells transfected with d/nPPARγ, but not in cells transfected with the pcDNA3 vector control, as compared to cells that were untransfected (Fig. 3A). More importantly, d/nPPARγ transfection also blocked each of the four metabolites’ anti-proliferative activity whereas pcDNA3 did not, again as compared to cells that were untransfected (Fig. 3B). In support of this last result, the anti-proliferative activity of the metabolites was inhibited in PC3 cells pretreated with the PPARγ antagonist, GW6992, as compared to cells treated with the drug’s vehicle (Fig. 3C). Finally, SDC-1 induction also appeared essential for the metabolites’ anti-proliferative activity. Cells that had their SDC-1 knocked down by transfection with SDC-1-specific siRNA were unresponsive (i.e. failed to stop proliferating in response) to the metabolites as compared to cells transfected with control siRNA or not transfected (Fig. 3D). Although 10,17-diHDHA and 7–17-HDHA were relatively weak activators of PPARγ (Fig. 2B), the effects of these metabolites on SDC-1 expression and proliferation were also sensitive to PPARγ inhibition (Fig. 3A–D). This suggests that a low threshold of receptor activation may be sufficient for PPARγ upregulation of the *sdc-1* gene which is consistent with effects of DHA on this pathway shown in previous studies [38].

## Discussion

Most of the PUFA oxygenases and metabolites that they make appear linked to the progression of prostate cancer because these metabolites promote the cells of this cancer to proliferate [15], [16], [21], [23], [24], [26], [27], [28], [30], [34], [50], [51], [52], [53]. 15-LOX-2 and its metabolites are outstanding exceptions to this rule: they appear linked to the suppression of prostate cancer in part because these metabolites inhibit proliferation [15], [19], [20], [22], [24], [25], [26]. Based on their in vitro activity and dominance as 15-LOX-2 metabolites, 15-HETE [24], [25], [26], [34], 15-hydroxy-eicostrienoic acid, and 15-hydroxy-eicosapentaenoic acid [35], [36] are candidate mediators of 15-LOX-2′s anti-proliferative effect. However, the low potency of these metabolites allows that products derived from other PUFA might be more potent and therefore more important in mediating the 15-LOX-2 effect. We find that members of the 17-series of DHA metabolites, 17-HpDHA, 17-HDHA, 7,17-diHDHA, and 10,17-diHDHA, inhibit the proliferation of androgen-independent (PC3 and DU145) and androgen-dependent (LNCaP) prostate cancer cells. The most potent of these, 17-HpDHA and 17-HDHA, significantly slowed proliferation at concentrations of ≥1 and 100 nM, respectively, and therefore are >1,000-fold more potent than the corresponding metabolites of AA; they also appear far more potent than the 15-LOX metabolites of EPA and GLA as reported in [35], [36]. The DHA metabolites clearly acted in a structural specific manner as evidenced by their decidedly different individual potencies and by their greater potencies than their counterparts in the 15-series of AA metabolites. We note that the activities of the 17-series of DHA metabolites found here do not exclude possibilities that their effects involve their further cellular metabolism to even more potent anti-proliferative products. We are just beginning to examine this issue.

Studies have shown that DHA suppresses the proliferation of prostate cancer cells including PC3 cells by a pathway that involves the activation of PPARγ, the binding of PPARγ to the SDC-1 promoter, the induction of SDC-1, and SDC-1-induced apoptosis [12], [38]:
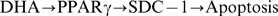



The DHA metabolites studied here stimulated PC3 cells to activate a PPARγ reporter, express SDC-1, and activate caspase-3, thus suggesting an additional important step in this pathway i.e the metabolism of DHA to more potent intermediates. Moreover, the PPARγ antagonist, GW6992, d/nPPARγ, and SDC-1 silencing blocked the DHA metabolites’ anti-proliferative action; d/nPPARγ also blocked their induction of SDC-1. The metabolites thus used the same signaling pathway as DHA to slow the proliferation of PC3 cells. This set of findings opens the possibility that the anti-proliferative effect of DHA is mediated at least in part through its metabolism by 15-LOX-2 to the 17-series of metabolites, particularly 17-HpDHA and 17-HDHA. There are, however, several problems with this scheme.

Studies disagree on the ability of PC3 cells to metabolize PUFA with some finding the cells make no [26] or very little [23], [24] 13-HODE and 15-HETE and others finding they make appreciable amounts of 13-HODE [15], [16] but little 15-HETE [15] even after exposure to high concentrations of LA or AA. Since 15-LOX-1 prefers LA to AA while 15-LOX-2 prefers AA to LA [14], [17], [18], [19], [20], these results indicate that PC3 cells have no or little 15-LOX-2 metabolizing activity, a result fully compatible with findings that these cells have 15-LOX-1 but little or no 15-LOX-2 message and protein [15], [24], [54]. In addition, the relative ability and specificity of the two human enzymes to use DHA as a substrate have not been defined although a 15-LOX-1 knock-down study in retinal pigment epithelial cells suggests that 15-LOX-1 but not 15-LOX-2 is responsible for metabolizing DHA to the 17-series of metabolites [55]. It is clear that the 17-series of DHA metabolites are made by various cell types in vitro and numerous tissue types in vivo [45], [55], [56], [57], [58]. However, the ability of malignant as well as normal prostate cells and tissues to make these metabolites and the contribution of 15-LOX-1 versus 15-LOX-2 to this is not known. We found that PC3 cells challenged with an anti-proliferative concentration (i.e. 100 µM) of DHA for 0.5–96 h converted only very small quantities (<0.003%) of it to 17-HDHA, 7,17-diHDHA, plus 10,17-diHDHA, as detected by selective ion-monitoring-MS (unpublished studies). These quantities seemed insufficient to slow proliferation. Faced with these findings, it might be profitable to consider other avenues by which prostate cancer could be subjected to these metabolites. Human prostate cancer juxtaposes with normal tissue. This normal tissue could provide the 17-series of DHA metabolites through the activity of 15-LOX-1, 15-LOX-2, or cytochrome P450 [59], [60]. 17-series DHA metabolites also form through auto-oxidation [61]; cultured neuroblastoma cells, for example, metabolize DHA to 17-HDHA and other cytotoxic DHA derivatives through auto-oxidation as well as 15-LOX-dependent pathways [56]. One or more of these alternative paths may be the means by which dietary n-3 PUFA ultimately act to reduce the mortality of prostate cancer [31].

In conclusion, we find that a series of 15-LOX-derived metabolites of DHA, particularly 17-HpDHA and 17-HDHA, are far more potent than their parent molecule or 15-LOX metabolites of other PUFA in inhibiting the proliferation of androgen positive and androgen negative human prostate cancer cell lines. Similar to their parent molecule, the DHA metabolites’ mechanism of action involves the PPARγ/SDC-1 apoptosis-signaling pathway. We propose that the prostate cancer-suppressing effect of dietary DHA is mediated in part by the conversion of DHA to one or more of these metabolites.

## References

[1] Norrish AE, Skeaff CM, Arribas GL, Sharpe SJ, Jackson RT (1999) Prostate cancer risk and consumption of fish oils: a dietary biomarker-based case-control study. Br J Cancer 81: 1238–1242. 10584888 10.1038/sj.bjc.6690835 PMC2374335

[2] Terry P, Lichtenstein P, Feychting M, Ahlbom A, Wolk A (2001) Fatty fish consumption and risk of prostate cancer. Lancet 357: 1764–1766. 11403817 10.1016/S0140-6736(00)04889-3

[3] Yang YJ, Lee SH, Hong SJ, Chung BC (1999) Comparison of fatty acid profiles in the serum of patients with prostate cancer and benign prostatic hyperplasia. Clin Biochem 32: 405–409. 10667474 10.1016/s0009-9120(99)00036-3

[4] Mamalakis G, Kafatos A, Kalogeropoulos N, Andrikopoulos N, Daskalopulos G, et al (2002) Prostate cancer vs hyperplasia: relationships with prostatic and adipose tissue fatty acid composition. Prostaglandins Leukot Essent Fatty Acids 66: 467–477. 12144866 10.1054/plef.2002.0384

[5] Freeman VL, Meydani M, Hur K, Flanigan RC (2004) Inverse association between prostatic polyunsaturated fatty acid and risk of locally advanced prostate carcinoma. Cancer 101: 2744–2754. 15495177 10.1002/cncr.20676

[6] Rose DP, Connolly JM (1991) Effects of fatty acids and eicosanoid synthesis inhibitors on the growth of two human prostate cancer cell lines. Prostate 18: 243–254. 2020620 10.1002/pros.2990180306

[7] Kobayashi N, Barnard RJ, Henning SM, Elashoff D, Reddy ST, et al (2006) Effect of altering dietary omega-6/omega-3 fatty acid ratios on prostate cancer membrane composition, cyclooxygenase-2, and prostaglandin E2. Clin Cancer Res 12: 4662–4670. 16899616 10.1158/1078-0432.CCR-06-0459 PMC3410648

[8] Berquin IM, Min Y, Wu R, Wu J, Perry D, et al (2007) Modulation of prostate cancer genetic risk by omega-3 and omega-6 fatty acids. J Clin Invest 117: 1866–1875. 17607361 10.1172/JCI31494 PMC1890998

[9] Larsson SC, Kumlin M, Ingelman-Sundberg M, Wolk A (2004) Dietary long-chain n-3 fatty acids for the prevention of cancer: a review of potential mechanisms. Am J Clin Nutr 79: 935–945. 15159222 10.1093/ajcn/79.6.935

[10] Berquin IM, Edwards IJ, Kridel SJ, Chen YQ (2011) Polyunsaturated fatty acid metabolism in prostate cancer. Cancer Metastasis Rev 30: 295–309. 22015690 10.1007/s10555-011-9299-7 PMC3865857

[11] Edwards IJ, Berquin IM, Sun H, O’Flaherty J T, Daniel LW, et al (2004) Differential effects of delivery of omega-3 fatty acids to human cancer cells by low-density lipoproteins versus albumin. Clin Cancer Res 10: 8275–8283. 15623603 10.1158/1078-0432.CCR-04-1357

[12] Hu Y, Sun H, Owens RT, Gu Z, Wu J, et al (2010) Syndecan-1-dependent suppression of PDK1/Akt/bad signaling by docosahexaenoic acid induces apoptosis in prostate cancer. Neoplasia 12: 826–836. 20927321 10.1593/neo.10586 PMC2950332

[13] Chung BH, Mitchell SH, Zhang JS, Young CY (2001) Effects of docosahexaenoic acid and eicosapentaenoic acid on androgen-mediated cell growth and gene expression in LNCaP prostate cancer cells. Carcinogenesis 22: 1201–1206. 11470750 10.1093/carcin/22.8.1201

[14] Kelavkar U, Lin Y, Landsittel D, Chandran U, Dhir R (2006) The yin and yang of 15-lipoxygenase-1 and delta-desaturases: dietary omega-6 linoleic acid metabolic pathway in prostate. J Carcinog 5: 9. 16566819 10.1186/1477-3163-5-9 PMC1440856

[15] Kelavkar UP, Cohen C, Kamitani H, Eling TE, Badr KF (2000) Concordant induction of 15-lipoxygenase-1 and mutant p53 expression in human prostate adenocarcinoma: correlation with Gleason staging. Carcinogenesis 21: 1777–1787. 11023533 10.1093/carcin/21.10.1777

[16] Spindler SA, Sarkar FH, Sakr WA, Blackburn ML, Bull AW, et al (1997) Production of 13-hydroxyoctadecadienoic acid (13-HODE) by prostate tumors and cell lines. Biochem Biophys Res Commun 239: 775–781. 9367845 10.1006/bbrc.1997.7471

[17] Brash AR, Jisaka M, Boeglin WE, Chang MS, Keeney DS, et al (1999) Investigation of a second 15S-lipoxygenase in humans and its expression in epithelial tissues. Adv Exp Med Biol 469: 83–89. 10667314 10.1007/978-1-4615-4793-8_13

[18] Wecksler AT, Kenyon V, Deschamps JD, Holman TR (2008) Substrate specificity changes for human reticulocyte and epithelial 15-lipoxygenases reveal allosteric product regulation. Biochemistry 47: 7364–7375. 18570379 10.1021/bi800550n PMC2603187

[19] Jack GS, Brash AR, Olson SJ, Manning S, Coffey CS, et al (2000) Reduced 15-lipoxygenase-2 immunostaining in prostate adenocarcinoma: correlation with grade and expression in high-grade prostatic intraepithelial neoplasia. Hum Pathol 31: 1146–1154. 11014584 10.1053/hupa.2000.16670

[20] Shappell SB, Manning S, Boeglin WE, Guan YF, Roberts RL, et al (2001) Alterations in lipoxygenase and cyclooxygenase-2 catalytic activity and mRNA expression in prostate carcinoma. Neoplasia 3: 287–303. 11571629 10.1038/sj.neo.7900166 PMC1505867

[21] Kelavkar UP, Parwani AV, Shappell SB, Martin WD (2006) Conditional expression of human 15-lipoxygenase-1 in mouse prostate induces prostatic intraepithelial neoplasia: the FLiMP mouse model. Neoplasia 8: 510–522. 16820097 10.1593/neo.06202 PMC1601466

[22] Suraneni MV, Schneider-Broussard R, Moore JR, Davis TC, Maldonado CJ, et al (2010) Transgenic expression of 15-lipoxygenase 2 (15-LOX2) in mouse prostate leads to hyperplasia and cell senescence. Oncogene 29: 4261–4275. 20514017 10.1038/onc.2010.197 PMC3042242

[23] Kelavkar UP, Nixon JB, Cohen C, Dillehay D, Eling TE, et al (2001) Overexpression of 15-lipoxygenase-1 in PC-3 human prostate cancer cells increases tumorigenesis. Carcinogenesis 22: 1765–1773. 11698337 10.1093/carcin/22.11.1765

[24] Bhatia B, Maldonado CJ, Tang S, Chandra D, Klein RD, et al (2003) Subcellular localization and tumor-suppressive functions of 15-lipoxygenase 2 (15-LOX2) and its splice variants. J Biol Chem 278: 25091–25100. 12704195 10.1074/jbc.M301920200

[25] Tang DG, Bhatia B, Tang S, Schneider-Broussard R (2007) 15-lipoxygenase 2 (15-LOX2) is a functional tumor suppressor that regulates human prostate epithelial cell differentiation, senescence, and growth (size). Prostaglandins Other Lipid Mediat 82: 135–146. 17164141 10.1016/j.prostaglandins.2006.05.022

[26] Hsi LC, Wilson LC, Eling TE (2002) Opposing effects of 15-lipoxygenase-1 and -2 metabolites on MAPK signaling in prostate. Alteration in peroxisome proliferator-activated receptor gamma. J Biol Chem 277: 40549–40556. 12189136 10.1074/jbc.M203522200

[27] Kelavkar UP, Cohen C (2004) 15-lipoxygenase-1 expression upregulates and activates insulin-like growth factor-1 receptor in prostate cancer cells. Neoplasia 6: 41–52. 15068670 10.1016/s1476-5586(04)80052-6 PMC1508629

[28] Shappell SB, Gupta RA, Manning S, Whitehead R, Boeglin WE, et al (2001) 15S-Hydroxyeicosatetraenoic acid activates peroxisome proliferator-activated receptor gamma and inhibits proliferation in PC3 prostate carcinoma cells. Cancer Res 61: 497–503. 11212240

[29] Subbarayan V, Krieg P, Hsi LC, Kim J, Yang P, et al (2006) 15-Lipoxygenase-2 gene regulation by its product 15-(S)-hydroxyeicosatetraenoic acid through a negative feedback mechanism that involves peroxisome proliferator-activated receptor gamma. Oncogene 25: 6015–6025. 16682954 10.1038/sj.onc.1209617

[30] Ghosh J, Myers CE (1997) Arachidonic acid stimulates prostate cancer cell growth: critical role of 5-lipoxygenase. Biochem Biophys Res Commun 235: 418–423. 9199209 10.1006/bbrc.1997.6799

[31] Amin M, Jeyaganth S, Fahmy N, Begin LR, Aronson S, et al (2008) Dietary habits and prostate cancer detection: a case-control study. Can Urol Assoc J 2: 510–515. 18953447 PMC2572247

[32] Astorg P (2005) [Dietary fatty acids and colorectal and prostate cancers: epidemiological studies]. Bull Cancer 92: 670–684. 16123006

[33] Williams CD, Whitley BM, Hoyo C, Grant DJ, Iraggi JD, et al (2011) A high ratio of dietary n-6/n-3 polyunsaturated fatty acids is associated with increased risk of prostate cancer. Nutr Res 31: 1–8. 21310299 10.1016/j.nutres.2011.01.002

[34] O’Flaherty JT, Rogers LC, Paumi CM, Hantgan RR, Thomas LR, et al (2005) 5-Oxo-ETE analogs and the proliferation of cancer cells. Biochim Biophys Acta 1736: 228–236. 16154383 10.1016/j.bbalip.2005.08.009

[35] Pham H, Banerjee T, Nalbandian GM, Ziboh VA (2003) Activation of peroxisome proliferator-activated receptor (PPAR)-gamma by 15S-hydroxyeicosatrienoic acid parallels growth suppression of androgen-dependent prostatic adenocarcinoma cells. Cancer Lett 189: 17–25. 12445673 10.1016/s0304-3835(02)00498-6

[36] Vang K, Ziboh VA (2005) 15-lipoxygenase metabolites of gamma-linolenic acid/eicosapentaenoic acid suppress growth and arachidonic acid metabolism in human prostatic adenocarcinoma cells: possible implications of dietary fatty acids. Prostaglandins Leukot Essent Fatty Acids 72: 363–372. 15850718 10.1016/j.plefa.2005.02.002

[37] Burdge GC, Wootton SA (2002) Conversion of alpha-linolenic acid to eicosapentaenoic, docosapentaenoic and docosahexaenoic acids in young women. Br J Nutr 88: 411–420. 12323090 10.1079/BJN2002689

[38] Edwards IJ, Sun H, Hu Y, Berquin IM, O’Flaherty JT, et al (2008) In vivo and in vitro regulation of syndecan 1 in prostate cells by N-3 polyunsaturated fatty acids. J Biol Chem 283: 18441–18449. 18450755 10.1074/jbc.M802107200 PMC2440608

[39] O’Flaherty JT, Thomas MJ (1985) Effect of 15-lipoxygenase-derived arachidonate metabolites on human neutrophil degranulation. Prostaglandins Leukot Med 17: 199–212. 3920680 10.1016/0262-1746(85)90107-6

[40] Butovich IA, Lukyanova SM, Bachmann C (2006) Dihydroxydocosahexaenoic acids of the neuroprotectin D family: synthesis, structure, and inhibition of human 5-lipoxygenase. J Lipid Res 47: 2462–2474. 16899822 10.1194/jlr.M600280-JLR200

[41] Butovich IA (2006) A one-step method of 10,17-dihydro(pero)xydocosahexa-4Z,7Z,11E,13Z,15E,19Z-enoic acid synthesis by soybean lipoxygenase. J Lipid Res 47: 854–863. 16391324 10.1194/jlr.D500042-JLR200

[42] Chen P, Vericel E, Lagarde M, Guichardant M (2011) Poxytrins, a class of oxygenated products from polyunsaturated fatty acids, potently inhibit blood platelet aggregation. Faseb J 25: 382–388. 20833872 10.1096/fj.10-161836

[43] Serhan CN, Gotlinger K, Hong S, Lu Y, Siegelman J, et al (2006) Anti-inflammatory actions of neuroprotectin D1/protectin D1 and its natural stereoisomers: assignments of dihydroxy-containing docosatrienes. J Immunol 176: 1848–1859. 16424216 10.4049/jimmunol.176.3.1848

[44] Dangi B, Obeng M, Nauroth JM, Teymourlouei M, Needham M, et al (2009) Biogenic synthesis, purification, and chemical characterization of anti-inflammatory resolvins derived from docosapentaenoic acid (DPAn-6). J Biol Chem 284: 14744–14759. 19324874 10.1074/jbc.M809014200 PMC2685656

[45] Gonzalez-Periz A, Planaguma A, Gronert K, Miquel R, Lopez-Parra M, et al (2006) Docosahexaenoic acid (DHA) blunts liver injury by conversion to protective lipid mediators: protectin D1 and 17S-hydroxy-DHA. Faseb J 20: 2537–2539. 17056761 10.1096/fj.06-6250fje

[46] Hong S, Gronert K, Devchand PR, Moussignac RL, Serhan CN (2003) Novel docosatrienes and 17S-resolvins generated from docosahexaenoic acid in murine brain, human blood, and glial cells. Autacoids in anti-inflammation. J Biol Chem 278: 14677–14687. 12590139 10.1074/jbc.M300218200

[47] Hu Y, Sun H, Owens RT, Wu J, Chen YQ, et al (2009) Decorin suppresses prostate tumor growth through inhibition of epidermal growth factor and androgen receptor pathways. Neoplasia 11: 1042–1053. 19794963 10.1593/neo.09760 PMC2745670

[48] Sun H, Berquin IM, Owens RT, O’Flaherty JT, Edwards IJ (2008) Peroxisome proliferator-activated receptor gamma-mediated up-regulation of syndecan-1 by n-3 fatty acids promotes apoptosis of human breast cancer cells. Cancer Res 68: 2912–2919. 18413760 10.1158/0008-5472.CAN-07-2305 PMC3686510

[49] Sun H, Hu Y, Gu Z, Owens RT, Chen YQ, et al (2011) Omega-3 fatty acids induce apoptosis in human breast cancer cells and mouse mammary tissue through syndecan-1 inhibition of the MEK-Erk pathway. Carcinogenesis 32: 1518–1524. 21771724 10.1093/carcin/bgr132 PMC3179420

[50] Hyde CA, Missailidis S (2009) Inhibition of arachidonic acid metabolism and its implication on cell proliferation and tumour-angiogenesis. Int Immunopharmacol 9: 701–715. 19239926 10.1016/j.intimp.2009.02.003

[51] Kelavkar UP, Hutzley J, McHugh K, Allen KG, Parwani A (2009) Prostate tumor growth can be modulated by dietarily targeting the 15-lipoxygenase-1 and cyclooxygenase-2 enzymes. Neoplasia 11: 692–699. 19568414 10.1593/neo.09334 PMC2697355

[52] Matsuyama M, Yoshimura R (2008) The target of arachidonic acid pathway is a new anticancer strategy for human prostate cancer. Biologics 2: 725–732. 19707453 10.2147/btt.s3151 PMC2727910

[53] Pidgeon GP, Lysaght J, Krishnamoorthy S, Reynolds JV, O’Byrne K, et al (2007) Lipoxygenase metabolism: roles in tumor progression and survival. Cancer Metastasis Rev 26: 503–524. 17943411 10.1007/s10555-007-9098-3

[54] Subbarayan V, Sabichi AL, Kim J, Llansa N, Logothetis CJ, et al (2004) Differential peroxisome proliferator-activated receptor-gamma isoform expression and agonist effects in normal and malignant prostate cells. Cancer Epidemiol Biomarkers Prev 13: 1710–1716. 15533897

[55] Calandria JM, Marcheselli VL, Mukherjee PK, Uddin J, Winkler JW, et al (2009) Selective survival rescue in 15-lipoxygenase-1-deficient retinal pigment epithelial cells by the novel docosahexaenoic acid-derived mediator, neuroprotectin D1. J Biol Chem 284: 17877–17882. 19403949 10.1074/jbc.M109.003988 PMC2719426

[56] Gleissman H, Yang R, Martinod K, Lindskog M, Serhan CN, et al (2010) Docosahexaenoic acid metabolome in neural tumors: identification of cytotoxic intermediates. Faseb J 24: 906–915. 19890019 10.1096/fj.09-137919 PMC2830131

[57] Poulsen RC, Gotlinger KH, Serhan CN, Kruger MC (2008) Identification of inflammatory and proresolving lipid mediators in bone marrow and their lipidomic profiles with ovariectomy and omega-3 intake. Am J Hematol 83: 437–445. 18429055 10.1002/ajh.21170

[58] Serhan CN, Chiang N, Van Dyke TE (2008) Resolving inflammation: dual anti-inflammatory and pro-resolution lipid mediators. Nat Rev Immunol 8: 349–361. 18437155 10.1038/nri2294 PMC2744593

[59] Fer M, Dreano Y, Lucas D, Corcos L, Salaun JP, et al (2008) Metabolism of eicosapentaenoic and docosahexaenoic acids by recombinant human cytochromes P450. Arch Biochem Biophys 471: 116–125. 18206980 10.1016/j.abb.2008.01.002

[60] VanRollins M, Baker RC, Sprecher HW, Murphy RC (1984) Oxidation of docosahexaenoic acid by rat liver microsomes. J Biol Chem 259: 5776–5783. 6232277

[61] VanRollins M, Murphy RC (1984) Autooxidation of docosahexaenoic acid: analysis of ten isomers of hydroxydocosahexaenoate. J Lipid Res 25: 507–517. 6234372

